# Arachnoiditis Ossificans in the Thoracic Spine With Associated Cyst and Syringomyelia: A Rare, Intraoperative Finding Complicating Dural Opening

**DOI:** 10.7759/cureus.16910

**Published:** 2021-08-05

**Authors:** David Bailey, Christine Mau, Elias Rizk, John Kelleher

**Affiliations:** 1 Neurological Surgery, Penn State Health Milton S. Hershey Medical Center, Hershey, USA

**Keywords:** arachnoiditis ossificans, syringomyelia, arachnoid cyst, arachnoiditis, spine

## Abstract

Arachnoiditis ossificans (AO) is a rare spinal pathology that develops because of bony metaplasia secondary to chronic inflammation. AO may present with debilitating myelopathy secondary to nerve root compression, making it distinct from spinal calcification commonly seen with aging. AO is extremely rare, having been reported less than 100 times, most commonly in the thoracic spine. Even rarer still, AO has been associated with syringomyelia and arachnoid cyst because of associated cerebrospinal fluid (CSF) flow disruption. In this report, we describe a case of AO that presented with right shoulder pain, right-hand numbness, and bilateral lower extremity fatigue who had syringomyelia and arachnoid cyst discovered on MRI imaging. When brought to the operating room for syrinx shunting and cyst fenestration the dural opening was complicated by severe calcification and a diagnosis of AO was made. The patient was treated with partial resection of the calcified plaques. Syringomyelia shunting was abandoned due to low volume. Post-operatively, the patient had improvement in their myelopathy though syrinx was still visualized on follow-up imaging. This report reviews the pathology, clinical and radiographic diagnosis, and treatment strategies for arachnoiditis ossificans.

## Introduction

Arachnoiditis ossificans (AO) is a rare spinal pathology first described in 1971 that is defined as bony metaplasia of the arachnoid membrane secondary to a chronic inflammatory state. Inflammation occurs secondary to spinal injury or manipulation though rarely it may be idiopathic. In addition, AO may be associated with the development of syringomyelia or an arachnoid cyst [1]. Even rarer still is the development of AO in the cervical spine, with only one reported case in the literature [2]. Treatment of AO typically involves a combination of medical and surgical management depending on clinical presentation and morphology. This report describes a case of AO that was discovered intraoperatively during a laminectomy with fenestration of an arachnoid cyst and shunting of a cervicothoracic syrinx. Following surgical treatment, the patient had improvement in their symptoms. This case represents an extremely rare and unexpected finding that may complicate an otherwise straightforward procedure. While arachnoiditis ossificans is rare, it is important to be familiar with this pathology if encountered.

## Case presentation

A 68-year-old female presented to the clinic with right shoulder pain, right-hand numbness, and muscle fatigue in her bilateral legs. The patient had a past medical history significant for T2-T3 diskectomy and fusion 14 years prior. Post-operative MRI three years after this procedure noted significant cord edema and arachnoid cysts causing cord compression and subsequent mild symptoms of thoracic back pain.

MRI imaging that was obtained upon patient presentation demonstrated a cervicothoracic syrinx from C4-T1 as well as a thoracic syrinx spanning from T3-T10, causing significant cord expansion. Post-contrast T1-weighted images demonstrated a subtle, thin layer of contrast enhancement surrounding the thoracic syrinx. There was reportedly a diagnosis of a spinal arteriovenous malformation (AVM) at an outside hospital, and given a family history of AVMs, further vascular imaging was obtained which proved negative. The patient’s symptoms began to worsen over the subsequent months and the patient elected for operative intervention. 

At this time, the patient was brought to the operating room for a T2-T9 laminectomy, arachnoid cyst fenestration, and possible placement of a syringo-arachnoid shunt. Laminectomy was completed and ultrasound was used to visualize the arachnoid cyst. However, the spinal cord and syringomyelia were not able to be visualized. Dissection into the dura was difficult secondary to significant calcification. The cranial and caudal extension was performed until past the calcified portions. The arachnoid cyst was punctured with a micro-nerve hook and fenestrated. The calcified dura was carefully removed and separated from the underlying cord.

**Figure 1:**
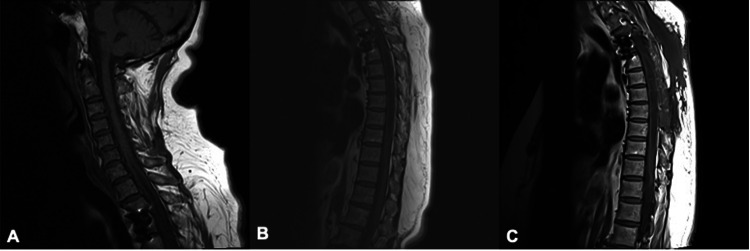
Preoperative and postoperative MRI imaging. (A) Preoperative T1 sequence of the cervical spine with hyperintensity posterior to the cord beginning at C7/T1. Syringomyelia may also be visualized. (B) Preoperative T1 sequence of the thoracic spine demonstrating hyperintensity posterior to the cord in the thoracic spine. Syringomyelia is also visualized. (C) Three-month post-operative T1 sequence of thoracic spine demonstrating decreased hyperintensity along the posterior cord relative to preoperative imaging. Syringomyelia may still be visualized.

Post-operative imaging demonstrated recurrent extramedullary cyst in the T3-T5 region as well as a decrease in size of the thoracic syrinx from the cervical segment to the T10 level (Figure 1). Post-operatively, the patient reported improved strength as well as full resolution of pain and numbness in the bilateral upper extremities. She continues to rehabilitate with physical and occupational therapy. Upon retrospective review of a CT abdomen performed years prior for unrelated symptoms, it appears the arachnoiditis ossificans was present prior to her thoracic discectomy.

## Discussion

AO is a rare spinal pathology that usually develops following spinal insult or manipulation. Inciting factors include prior surgery, infection, subarachnoid hemorrhage, lumbar epidural injections, oil-based myelography contrast agents, chemical irritation, and rarely may be idiopathic [3,4]. There is no clear temporal relationship between initial spinal insult and the development of AO [3]. As a result of chronic inflammation, dural adhesions and scar tissue can form which encapsulate nerve roots and disrupt cerebrospinal fluid (CSF) flow. Rarely, this chronic inflammation may progress to AO. Histologically, this presents as bony metaplasia of the meninges that is indistinguishable from normal bone [5]. Importantly, AO is distinct from the spinal calcification commonly seen with aging because of its ability to cause debilitating myelopathy [6].

AO is extremely rare, having been reported in the literature less than 100 times [7]. In their 2004 review of 47 cases, Domenicucci et al. reported cases in patients aged 22-71 years old with a mean of 53.7 years old [3]. More recent case reports have described cases in patients as young as 13 and 14 years old [8,9]. AO develops in the thoracic cord 66% of the time and the lumbar region 24% of the time [10]. Only one instance has been reported in the cervical spine [2].

AO typically presents with progressive neurologic degeneration secondary to nerve root compression [5]. The development of symptoms is related to calcification and compression of the spinal cord. Occasionally, syringomyelia and arachnoid cyst formation may be associated with AO as a result of disruption to CSF flow. This has been demonstrated in 12 cases of AO, including our own [1,3,4,11-17] (Table 1). Syringomyelia developed in the thoracic cord for all previous cases in close relationship to the ossification, similar to our own, who also developed a cervical syrinx.

**Table 1:** Arachnoiditis ossificans with syringomyelia. *Shunt placed in follow-up surgery after the return of symptoms with moderate improvement in symptoms. AO: Arachnoiditis ossificans

Author / Year	Year	Age / Sex	Syrinx Level	AO level	Procedure	Outcome
Kahler et al. [11]	1990	62/M	C7-T9	T2-T11	Thoracic laminectomy, syrinx shunt placement, no plaque removal	Clinical improvement, decreased syrinx size
Slavin et al. [12]	1999	54/F	T7-L3	T6-T9	T9-T11 laminectomy, syrinx shunt placed, plaque removal	Clinical improvement, decreased syrinx size
Revilla et al. [2]	1999	68/F	Cervicomedullary junction-conus medullary	T5-T9	T2-T5, removal of plaque	Clinical improvement
Domenicucci et al. [3]	2004	63/F	T2-T11	T2-T11	T3-T10 laminectomy, no plaque removal	Clinical improvement
﻿Papavlasopoulos et al. [13]	2007	30 /M	T10-L2	Lower thoracic / Lumbar	T8-T10 laminectomy, no plaque removal	No clinical improvement
Ibrahim et al. [1]	2010	36/ M	T3	T4-T8	T4-T7 laminectomy, plaque removal	Clinical improvement, decreased syrinx size
Singh et al. [14]	2011	81/F	T6-conus	T6-T8 and caudally	T6-T8 laminectomy, plaque removal	Clinical improvement, decreased syrinx size
Hasturk et al. [15]	2013	48/F	T6-T12	T9-T12	T9-T11 laminectomy, plaque removal	Clinical improvement
Opalak et al. [16]	2015	62/M	T6-conus	T5-T6-conus	T11-L2 laminectomy, shunt placement, plaque removal	Clinical improvement
Wang et al. [17]	2019	41/F	T6-T11	T5-T12	T6-T8 laminectomy, plaque removal	No clinical improvement
Wang et al. [4]	2019	70/F	Thoracic spine	T5-T11	T5-T8 laminectomies, plaque removal // shunt placement*	Clinical improvement
Our case	2021	68/F	C4-T1, T3-T10	T1-L2	T2-T9 laminectomy	Clinical improvement

In cases of AO with secondary pathologies such as syrinx or cyst, ossification was identified pre-operatively in eight of 12 cases. In a case presented by Slavin et al. the diagnosis of AO was made intraoperatively, due to failure of identification on MRI and no CT on record [12]. This is similar to our own case, where the neurologic symptoms were attributed to findings of a cyst and syrinx on MRI. MRI findings of AO are subtle, usually hyperintense on T1-weighted images and hypo or hyperintense on T2-weighted sequences [18]. The study of choice to identify AO is a nonenhanced CT scan because of its ability to detect ossified plaques. In our case, as with Slavin et al., a dedicated CT scan was not obtained due to the presence of pathology that explained the patient’s symptoms.

Arachnoiditis ossificans are commonly classified using the criteria laid out by Domenicucci et al., which considers morphology, clinical manifestation, and therapy [3]. This system separates AO into three categories, semicircular (I), circular (II) and ossification encapsulating the caudal fibers (III). Type I is commonly found in the thoracic spine, type II in the thoracic and lumbar region, and type III in the lumbar spine. Type I and II patients are typically amenable to surgical resection, while type III patients typically have less severe symptomology and may be treated medically [4]. Therefore, the patient presented here may be classified as Type I.

AO's treatment strategies have not been well-established and are typically decided on a case-by-case basis as determined by clinical presentation and morphology [19]. Medical treatment may be applied in more mild cases or where resection is determined not to be possible, often consisting of anti-inflammatories, analgesics, and neuroleptic medications depending on symptomology. Surgical treatment involving decompression and resection is a technically challenging procedure with variable outcomes and should be deferred with minor symptomology due to the risk of worsening injury. A review by Bagley et al. demonstrated 60% of patients improved, 30% were unchanged, and 6% worsened postoperatively. Patients with lumbar AO who were operated on had improved outcomes 65% of the time versus 58% of the time in thoracic AO, which may reflect overall more minor symptomology for lumbar AO [19].

In previous cases of AO with a concomitant syrinx, the treatment is variable. It is unclear whether directly treating the syringomyelia offers any clinical benefit. Several cases with syrinx aspiration and placement of CSF shunt resulted in the collapse of the syrinx with clinical improvement [11,12]. Only one of these cases involved plaque removal [12]. However, clinical improvement and even decreased syrinx size have been observed when myelotomy or shunting of the syrinx was not performed [1,14]. In our case, shunt placement was attempted but was abandoned because the syrinx was of low volume. Clinical improvement was observed despite the persistence of the syrinx. This contrasts with a case presented by Wang et al. where the patient initially experienced clinical improvement following plaque removal without shunting. However, after worsening of symptoms a recurrent syringomyelia was discovered and a shunt was placed with moderate effect [4].

## Conclusions

Arachnoiditis ossificans is an infrequent cause of myelopathy, most commonly secondary to a previous spinal insult. AO is best diagnosed using nonenhanced CT imaging though it may also be discovered intraoperatively during correction of associated pathology, including syringomyelia and arachnoid cyst. Treatment of AO is variable based on symptoms and morphology though it often includes a combination of surgical and medical management. In patients with associated syringomyelia, it is unclear whether treatment of the secondary pathology is necessary to improve outcomes.
